# Successful disinfection of femoral head bone graft using high hydrostatic pressure

**DOI:** 10.1007/s10561-017-9678-6

**Published:** 2017-12-20

**Authors:** Michiel A. J. van de Sande, Judith V. M. G. Bovée, Mark van Domselaar, Marja J. van Wijk, Ingrid Sanders, Ed Kuijper

**Affiliations:** 1 Department of Orthopaedic Surgery, Leiden University Medical Center, Leiden, Zuid-Holland The Netherlands; 2 Department of Pathology, Leiden University Medical Center, Leiden, Zuid-Holland The Netherlands; 3 Research Department, TDI-BV, Wageningen, Gelderland The Netherlands; 4 Medical Department, BISLIFE Foundation, Leiden, Zuid-Holland The Netherlands; 5 Department of Medical Microbiology, Leiden University Medical Center, Leiden, Zuid-Holland The Netherlands

**Keywords:** Disinfection, Bone graft, Hydrostatic pressure, Microorganisms

## Abstract

The current standard for sterilization of potentially infected bone graft by gamma irradiation and thermal or chemical inactivation potentially deteriorates the biomechanical properties of the graft. We performed an in vitro experiment to evaluate the use of high hydrostatic pressure (HHP); which is widely used as a disinfection process in the food processing industry, to sterilize bone grafts. Four femoral heads were divided into five parts each, of which 16 were contaminated (in duplicate) with 10^5^–10^7^ CFU/ml of *Staphylococcus epidermidis*, *Bacillus cereus*, or *Pseudomonas aeruginosa* or *Candida albicans*, respectively. Of each duplicate, one sample was untreated and stored similarly as the treated sample. The remaining four parts were included as sterile control and non-infected control. The 16 parts underwent HHP at the high-pressure value of 600 MPa. After HHP, serial dilutions were made and cultured on selective media and into enrichment media to recover low amounts of microorganism and spores. Three additional complete femoral heads were treated with 0, 300 and 600 MPa HHP respectively for histological evaluation. None of the negative-control bone fragments contained microorganisms. The measured colony counts in the positive-control samples correlated excellent with the expected colony count. None of the HHP treated bone fragments grew on culture plates or enrichment media. Histological examination of three untreated femoral heads showed that the bone structure remained unchanged after HHP. Sterilizing bone grafts by high hydrostatic pressure was successful and is a promising technique with the possible advantage of retaining biomechanical properties of bone tissue.

## Introduction

Sterilization of bone and soft tissue allograft at present is mainly performed using extracorporeal irradiation or autoclaving, generally followed by freeze-dried preservation in a bone bank (Diehl et al. 2005). However, sterilization by gamma irradiation and thermal or chemical inactivation of allografts and other biomaterials, considered for tissue regeneration and reconstruction, is associated with deterioration of the mechanical, physical and biological properties of the bony implant (Barth et al. 2011; Kaminski et al. 2012).

High hydrostatic pressure (HHP), successfully used in the food processing industry to disinfect and prolong expiration dates, has been proposed as a new entity for bone graft disinfection and preservation. In addition, HHP could be an alternative for irradiation and reimplantation of resected bone, after bone tumor resection (Diehl et al. 2003; Weber et al. 2008; Naal et al. 2005). The hypothesized additional advantage of HHP in the sterilization of bone graft would be the potential to retain biomechanical properties of the graft (Diehl et al. 2006; Naal et al. 2008). In 2007 Diehl et al. were the first to demonstrate that HHP can effectively devitalize and sterilize bone grafts, cartilage and tendon in vitro while leaving the tissues’ mechanical properties unimpaired, thus allowing for reimplantation of the resected tissue (Diehl et al. 2007). In addition, the bacteriostatic characteristics of HHP in contaminated and infected bone and implants at 300–600 MPa were reported, although no complete sterilisation of infected bone material could be achieved (Gollwitzer et al. 2009). To our knowledge, no other study groups have published on the use of HHP in bone graft sterilization. To explore further possible use in biomedical practice, we were interested in the application of HHP technology to sterilize or disinfect bone grafts. The first step allowing re-implantation of bone graft is to assess the sterilization process of bone grafts using the HHP process. Hereafter long-term preservation, structural integrity and biomechanical properties of the bone graft could be thoroughly assessed in further studies.

Our aim was to explore the sterilising properties of HHP treatment for bone graft in an in vitro experiment. We hypothesize that HHP treatment of contaminated bone graft results in a minimal log 4–5 reduction of the amount of induced bacterial contamination. We chose four commonly cultured microorganisms representative for different groups of microorganisms, found as contaminants of bone grafts used in the Netherlands (Bonebank, BISLIFE, Leiden, The Netherlands).

## Materials and methods

**Fig. 1:**
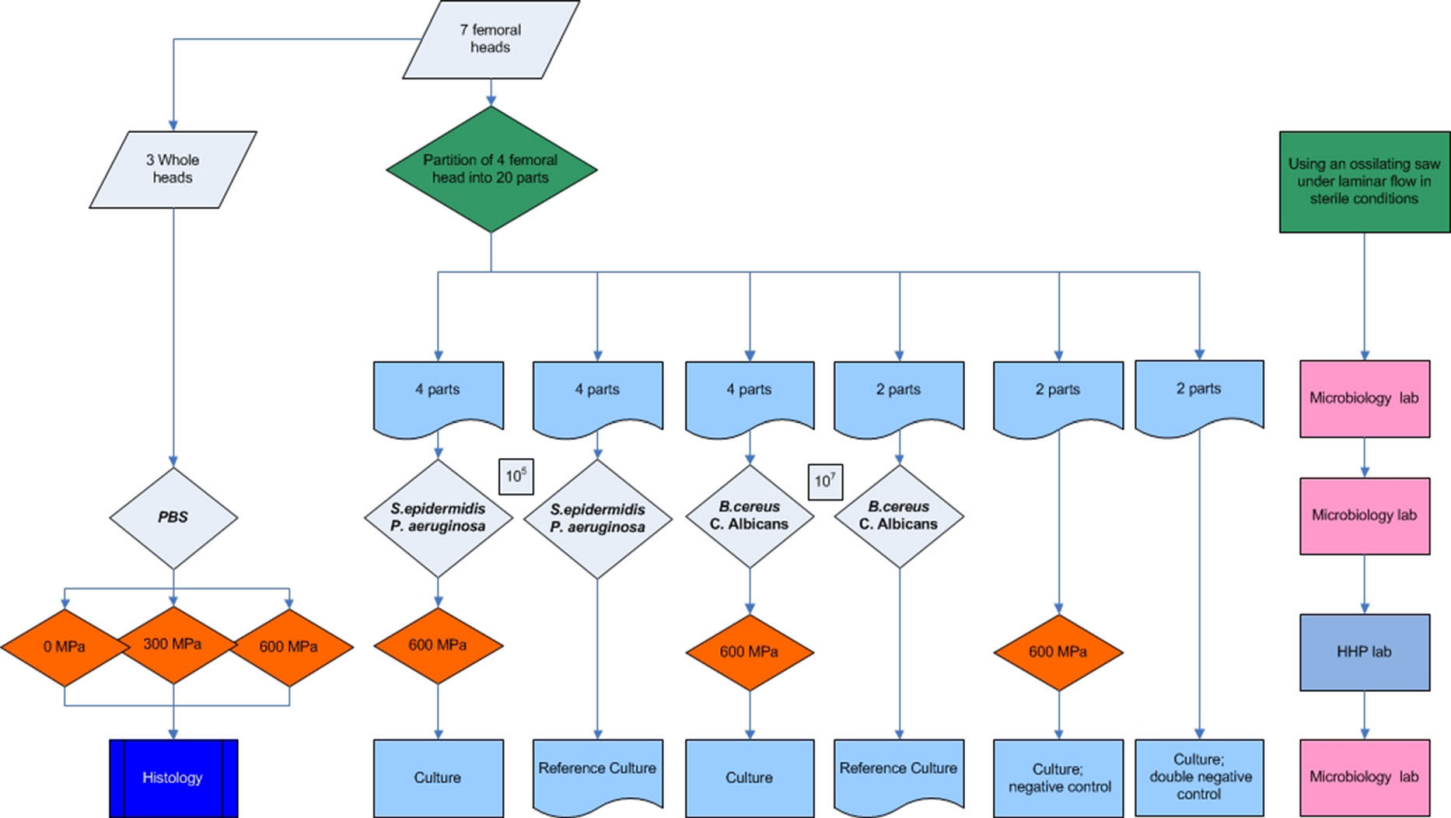
Flowchart for materials and methods

Seven fresh frozen femoral heads, that were unsuitable for transplantation for reasons other than infectious disease (Bonebank BISLIFE, Leiden The Netherlands), were selected. Donors and femoral heads were cultured by standard procedures (van de Pol et al. 2007) (swab culture of the surface of the whole femoral head) and all were found negative. Using an oscillation saw in the surgical theatre under down flow and sterile circumstances, four femoral heads were divided into 5 equal parts each (Fig. 1). They were again packaged using a sterile container and transported to the department for clinical microbiology, in a cooled box. Sixteen parts were contaminated in a sterile environment and subsequently sealed in a plastic bag containing 100 ml of phosphate-buffered saline (PBS) with 10^5−7^ CFU/ml of *Staphylococcus epidermidis* (ATCC 14990), *Bacillus cereus* (ATCC 14579), *Pseudomonas aeruginosa* (ATCC 27853) and *Candida albicans* (ATCC 10231), in duplicate, respectively (Fig. 1). As controls, four parts were not contaminated but directly packaged in 100 ml of the same phosphate-buffered saline (PBS); pH 7.5, in a sealed sterile plastic bag.

### Bacterial infection, dilution series and cultures

To obtain a starting concentration of 10^5^ CFU/ml of *S. epidermidis* and *P. aeruginosa*, 1 ml of a concentration of 10^7^ CFU/ml was diluted in 99 ml Phosphate buffered salt (PBS). The concentration of 10^7^ CFU/ml was estimated by McFarland and confirmed by culturing serial dilutions onto 5% sheep blood agar plates (SBA; bioMérieux, Marcy l’Etoile, France). Each contaminated femoral head was contaminated with a total volume of 100 ml.

For *C. albicans* and *B. cereus*, each sample was contaminated with 10^7^ CFU/ml. Similar as described above; a starting concentration was made using a McFarland dilution and confirmed by control cultures.

All contaminated bags and controls were labelled, vacuum-sealed in sterile bags and preserved and transported at cool conditions (± 4 °C) to undergo HHP at a secondary location, at Tournois Dynamic Innovations (TDI BV) Helmond, The Netherlands. The mechanism and use of HHP to disinfect bone and soft tissue graft was earlier described Diehl et al. (2007). Its mechanism relies on the cell disruptive properties of HPP while not affecting the extra cellular matrix or bone scaffold. To ensure the sterile containment and packaging, 150 × 200 mm 170 micron, plastic bags (Hevel Super Export Plus) were used. The plastic bags were sterilized by gamma irradiation. Pascalisation followed at TDI the next day, for 30 min at 600 MPa or for 30 min at 300 MPa in a Hiperbaric 55 (one whole femoral head only). Temperature of the process water before the pressure increase was 14–15 °C and the samples went in at 7 °C. The Adiabatic temperature rise caused by the increased pressure within the vessel was calculated using the following formula; for pure organic substances (Cp Å 2 kJ/kg K) the adiabatic temperature rise is often approximated by DTad = DHr/2 = 3 °C/100 MPa increase in pressure. Maximum temperature therefore never exceeded over 33 °C at 600 MPa.

After HHP, the samples were returned to the microbiology department in the LUMC under cooled circumstances at 4 °C within 24 h after HHP. All contaminated bags and controls were opened and cultured in a sterile environment. One ml of suspension was diluted in PBS (10^6^–10^5^–10^4^–10^3^–10^2^) and 50 µl of the mixture was cultured on SBA plates. From the undiluted samples 200 µl was plated out. All plates were incubated for 48 h at 37 °C. Additionally, the femoral head fragment was inoculated in an enrichment medium consisting of 100 ml liquid Brain Hart Infusion (BHI) broth. After 48 h incubation at 37 °C, BHI was sub-cultured to SBA, cysteine lactose electrolyte deficient agar (CLED; bioMérieux, Marcy l’Etoile, France) and Sabouraud agar (SAB; bioMérieux, Marcy l’Etoile, France) to examine growth of bacteria and yeasts within the bone graft.

### Histological evaluation

Three additional intact femoral heads were not contaminated but underwent the same protocol as the contaminated samples. The three femoral heads were packaged into a sterile environment into a plastic bag filled with 100 ml PBS and vacuum-sealed. One was left untreated, the other two underwent either HHP with 300 or 600 MPa and all were stored for 5 days at cool temperature (around 4 °C). The femoral heads were then cut in half and the central part of the femoral head was fixed in formalin, decalcified using formic acid, and embedded in paraffin. Four µm sections were stained with haematoxylin and eosin. All were standard procedures.

## Results and discussion

### Microbiology

**Fig. 2:**
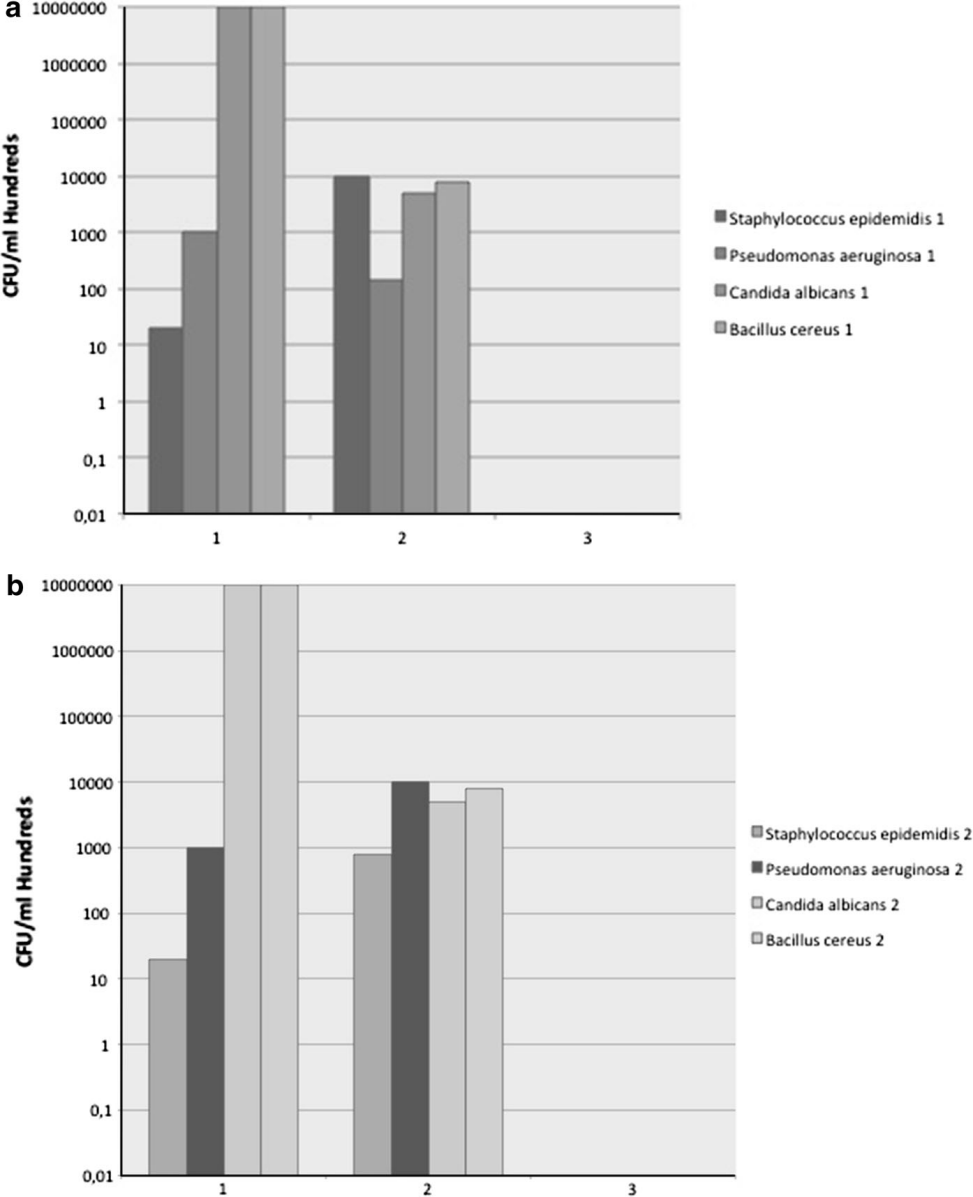
Test and retest (**a**, **b**) showing no growth for bacteria (*S. epidermidis*, *P. aeruginosa* and *B. cereus*) and yeast (*C. albicans*) after 600 MPa treatment. 1. Real starting concentration (CFU/ml in hundreds), 2. Concentration after transport without HHP (CFU/ml in hundreds), 3. Concentration after transport and 600 MPa HHP (CFU/ml in hundreds)

Both *S. epidermidis* and *P. aeruginosa* could not be cultured after HHP at 600 MPa. Starting with a concentration of 10^5^ this resulted in a log 5 reduction. Similarly, we found a log 7 reduction for *B. cereus* and *C. albicans* (Fig. 2a, b). The control of the starting concentration confirmed that the administered concentration was correct. The control bags that did not undergo HHP treatment, showed bacterial growth as expected (Fig. 2a, b). The concentration after transport to and from the HHP facility was somewhat lower than the starting concentration. Sample no. 9 was contaminated with a gram-positive rod, not belonging to the genus Bacillus which was therefore considered contamination during experimental protocol.

### Histology

**Fig. 3:**
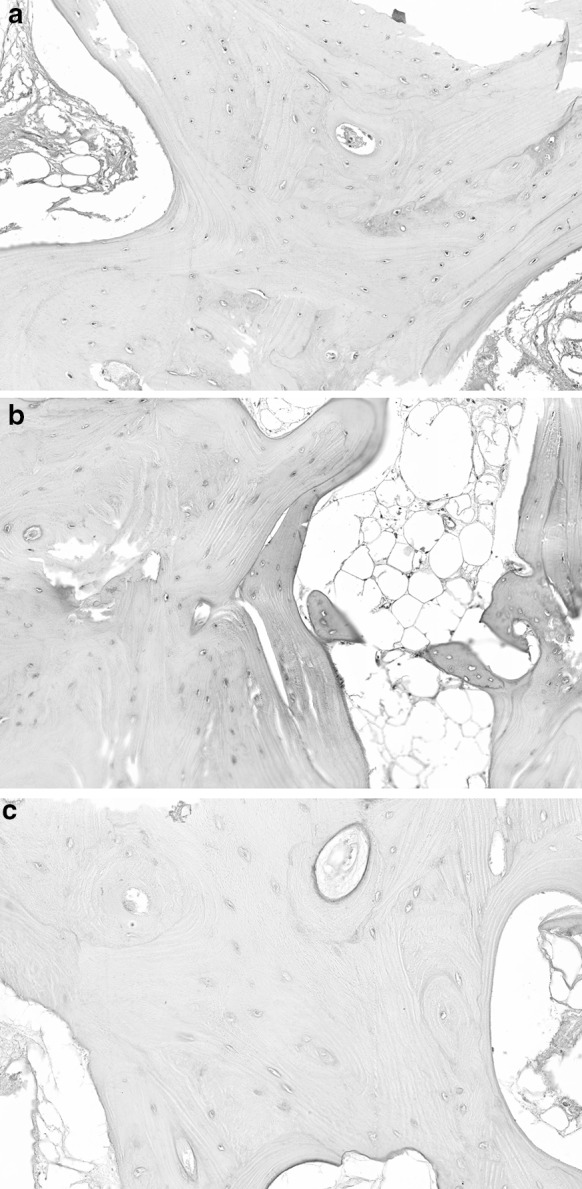
**a** Femoral head number 1 fully processed and conserved but without HHP treatment, H&E staining shows intact structure of the bone and vital osteocytes, **b** femoral head number 2 after 300 MPa HHP, H&E staining shows intact structure of the bone with some vital osteocytes, **c** femoral head number 3 after 600 MPa HHP, H&E staining shows intact structure of the bone with lack of nuclear staining of osteocytes

Haematoxylin and eosin staining of the three intact femoral heads revealed that microscopically the bone structure remained intact after HHP treatment (Fig. 3). There seemed to be a decrease in nuclear staining of the osteocytes after HHP treatment; in the untreated bone vital osteocytes were easily identified, after 300 MPa vital osteocytes were still present but in the treated bone with 600 MPa the lacunae appeared empty.

Contamination of bone tissue harvested in living donors or in a post-mortem explantation setting is estimated at 10–30% of all grafts, predominantly with *S epidermidis* (Schubert et al. 2012; Journeaux et al. 1998; Mathijssen et al. 2013; Sommerville et al. 2000). Although these high contamination rates underline the importance of reliable bone graft disinfection after harvesting, the risk for disease transmission through bone graft implantation remains extremely low (van de Pol et al. 2007; Sommerville et al. 2000; Tomford et al. 1990; Chiu et al. 2004). Graft disinfection with use of antibiotic solution is considered as insufficient (Deijkers et al. 1996). Therefore radiation treatment is commonly advised to control exogenous contamination during retrieval procedure and graft processing (Deijkers et al. 1996). However, radiation is reported to be deleterious to the biomechanical properties of bone graft (Mitchell et al. 2004). This negative influence of radiation on the structural integrity and biomechanical properties of bone grafts may be reduced by decreasing the radiation dosage to 15 kGy, but new reliable disinfection protocols providing optimal biomechanical properties of bone graft seem appropriate (Nguyen et al. 2013).

High hydrostatic pressure (HHP) at present is widely used in the food industry as it offers multiple opportunities for pasteurization, enzyme inactivation and freezing of foods utilizing the HHP induced pressure shift (San Martín et al. 2002). By changing the absolute pressure, hold time of the pressure, temperature and or chemical environment, the induced molecular changes can be either permanent or reversible. In general, a pressure-transferring medium, usually water, is necessary to provide uniform or isotropic transmission of pressure throughout a mass independent of size, shape, and composition. At higher pressures (300–600 MPa) most microorganisms and spores are reported to be (partially) eliminated (Knorr 1999). HHP even inactivates mycobacteria, spores of Bacillus species and certain enveloped and non-enveloped viruses (de Souza et al. 2013; Olivier et al. 2011; Kishida et al. 2013). The anti-microbial effect of HHP is well understood in food preservation but clinical use remains limited.

We found that HHP resulted in a significant reduction of the amount of bacteria and yeasts in contaminated femoral heads, which would make HHP suitable for disinfection of bone graft tissues to be used in (orthopaedic) surgery. We demonstrated that HHP reduced the amount of bacteria (*S. epidermidis*, *P. aeruginosa* and *B. cereus*) and yeast (*C. albicans*) by at least log 5. These results are comparable to the disinfection properties of radiation treatment of bone grafts with 15–25 kGy (Nguyen et al. 2008, 2013). Though we achieved complete sterilization and fulfil to the requirements of the European Pharmacopoeia, we still consider the experiments as a pilot study. The next step is to enlarge our study with inclusion of other types of grafts and additional but different microorganisms.

Our results are in contrast to the findings of Gollwitzer et al. (2009), who reported significant failure rates after HHP disinfection of contaminated bone. Although Gollwitzer found a clear effect using 300 MPa on *S. aureus* contaminated stainless steel screws, no significant effect was found on artificially infected bone grafts harvested from patients with aseptic osteoarthritis. These findings are in contrast with our results, which may be explained by a baroprotective effect of the surrounding inflamed bone in some of the samples tested by Gollwitzer. This hypothesis is supported by their observation that various bone specimens could be completely disinfected, whereas others proved resistant to treatment with unaffected bacterial growth (Gollwitzer et al. 2009). Additionally, they underlined the possibility of barosensitive and barotolerant microorganisms and strains, which was also postulated by Alpas et al. (1999). Further evaluation of susceptibility of different microorganisms, strains and spores is therefore warranted.

We also demonstrated that the bone structure remains microscopically unaltered after HHP. Additional micro-CT evaluation after HHP treatment in the future will possibly allow for more detail on the structural integrity of bone grafts after HHP. In addition, we report that viable osteocytes seem to decrease in numbers with increased pressure of HHP, which was also reported previously by others (Naal et al. 2008; Diehl et al. 2008). However, the number of femoral heads in our study is too low to draw definitive conclusions. Also, we cannot rule out that the clinical history of these selected femoral heads may have included osteoarthritis, osteonecrosis or fracture. These three underlying diseases may have influenced the initial viability of the osteocytes and may have altered bone architecture.

The ultimate balance between the high pressure necessary to disinfect the bone graft and maximum pressure tolerated by the osteocytes needs further attention. Recent publications have underlined the important role of osteocyte apoptosis in bone remodelling through RANKL expression and sclerostin secretion, inducing osteoclastogenesis and osteoclastic bone resorption (Graham et al. 2013; Pajevic et al. 2010).

The osteoconductive and osteoinductive properties of HHP treated bone graft were indirectly explored earlier, using HHP treated bone as scaffold for seeded viable osteoblast-like cells prepared from donor bones (Schauwecker et al. 2011). Schauwecker et al. reported that independent of the applied HHP protocol 74% of the seeded cells adhered to the bone matrix. They anticipated that HHP treatment up to 600 MPa causes no alterations in bone matrix that could impair the osteoconductivity of the graft. Their conclusions again warrant further exploration of the osteoconductive, but also osteoinductive potential of HHP treated bone tissues.

One of the limitations of our study is that only four different microorganisms were used. Additional tests for less frequent contaminants are proposed. The ultimate effect on the survival of spores of the Bacillus was not fully investigated as we only cultured up to 92 h. Moreover, our study only focuses on contaminations occurring before storage of the bone grafts. More information is warranted on possible late contamination of the bone graft during transport and storage just before use in the surgical theatre. Additionally, as this disinfection technique possibly allows storage of the bone graft in temperatures above zero degrees, direct use during surgery would be possible, but the effect of cooled preservation of bone graft on the bacterial disinfection and the vitality of the allografts osteocytes need further evaluation. In addition, the final concentration of bacteria and yeasts after transport were somewhat lower than the starting concentration. This can be explained by the duration of the complete experiment and the effect of low temperature on the microorganisms. However, the contamination concentrations were sufficiently high to evaluate our hypothesis.

## Conclusions

In conclusion, this report presents promising initial results for the use of HHP in the disinfection of bone grafts. It underlines the importance of further pre-clinical evaluation of its efficacy in other microorganisms and strains as well as its influence on bone remodelling and the structural integrity of bone grafts.

## Abbreviations

CFU: Colony forming units
McF: MacFarland
HPP: High pressure processing
BHI: Brain heart infusion
PBS: Phosphate-buffered saline
SAB: Sabouraud medium
SBA: 5% sheep blood agar medium
